# Impact of Rapid Pacing Time on Myocardial Injury in Transcatheter Aortic Valve Implantation for Non–End-stage Renal Disease Patients

**DOI:** 10.1016/j.cjco.2025.08.011

**Published:** 2025-08-28

**Authors:** Keisuke Matsuo, Takahide Arai, Mitsunobu Nagai, Yuto Hori, Hiroki Hoya, Yodo Gatate, Akihiro Yoshitake, Shintaro Nakano

**Affiliations:** a Department of Cardiology, Saitama Medical University, International Medical Center, Saitama, Japan; b Department of Cardiovascular Surgery, Saitama Medical University, International Medical Center, Saitama, Japan

**Keywords:** periprocedural myocardial injury, rapid pacing, renal dysfunction, transcatheter aortic valve implantation, troponin

## Abstract

**Background:**

Periprocedural myocardial injury (PMI) is a concern in transcatheter aortic valve implantation (TAVI), with rapid pacing (RP) suspected to be a contributing factor. PMI is defined by elevated troponin levels. In this study we determined the net effect of RP on PMI after excluding patients with severe renal dysfunction by evaluating troponin elevation after TAVI.

**Methods:**

We included 137 patients who underwent TAVI between September 2023 and January 2025. The association between renal function and cardiac troponin T (cTnT) level was investigated. Patients were categorized according to the RP time (RPT) to investigate its association with cTnT elevation, PMI, and short-term outcomes. The 100 patients with an estimated glomerular filtration rate (eGFR) ≥ 30 mL/min per 1.73 m^2^ were divided into 2 groups: short (< 18 seconds, n = 49) and long (≥ 18 seconds, n = 51) RPT. The primary endpoint was PMI/troponin levels, whereas the secondary endpoints were 30-day all-cause death and major adverse cardiovascular events (MACE).

**Results:**

The eGFR inversely correlated with cTnT levels (*P* < 0.001). The long RPT group had significantly higher cTnT values (*P* = 0.026) and PMI rates (14.2% vs 33.3%, *P* = 0.034) vs the short RPT group. The 30-day prognosis did not differ between the short and long RPT groups. Patients with PMI exhibited a trend toward higher MACE (*P* = 0.051) vs those without PMI. ΔcTnT independently predicted 30-day MACE (*P* = 0.043).

**Conclusions:**

A longer RPT significantly increased troponin levels, indicating PMI, which was associated with worse short-term prognosis of cardiovascular events. However, other factors, such as renal dysfunction, rather than only longer RPT, are also associated with increased troponin level.

## Central Illustrations

**Figure:**
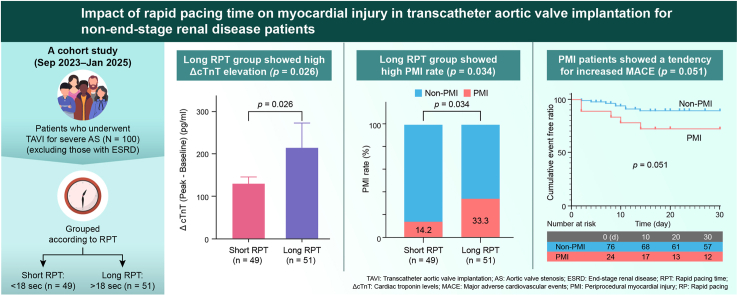

## Résumé

**Contexte:**

Les lésions myocardiques périopératoires (LMPO) constituent un sujet de préoccupation dans le cadre de l'implantation valvulaire aortique par cathéter (IVAC), la stimulation rapide (SR) étant soupçonnée d'être un facteur contributif. Les LMPO se caractérisent par des taux élevés de troponine. Cette étude a déterminé l'effet net de la SR sur les LMPO après avoir exclu les patients présentant une insuffisance rénale sévère, en évaluant l'élévation du taux de troponine après IVAC.

**Méthodologie:**

Nous avons inclus 137 patients ayant subi une IVAC entre septembre 2023 et janvier 2025. L'association entre la fonction rénale et le taux de troponine T cardiaque (TnTc) a été étudiée. Les patients ont été classés en fonction de la durée de la SR (DSR) afin d'étudier son association avec l'élévation de la TnTc, les LMPO et les résultats cliniques à court terme. Les 100 patients présentant un débit de filtration glomérulaire estimé (DFGe) ≥ 30 ml/min/1,73 m^2^ ont été divisés en deux groupes : DSR courte (< 18 s, n = 49) et longue (≥ 18 s, n = 51). Le critère d'évaluation principal était les LMPO/élévation des taux de troponine, tandis que les critères d'évaluation secondaires étaient la mortalité toutes causes confondues à 30 jours et les événements cardiovasculaires indésirables majeurs (ECIM).

**Résultats:**

Le DFGe était inversement corrélé aux taux de TnTc (p < 0,001). Le groupe avec longue DSR présentait des valeurs de TnTc (*p* = 0,026) et des taux de LMPO (14,2 % contre 33,3 %, *p* = 0,034) significativement plus élevés que le groupe avec courte DSR. Le pronostic à 30 jours ne différait pas entre les groupes DSR courte et longue. Les patients présentant une LMPO avaient tendance à présenter un taux d'ECIM plus élevé (*p* = 0,051) que ceux sans LMPO. Le ΔTnTc prédisait de manière indépendante les ECIM à 30 jours (*p* = 0,043).

**Conclusions:**

Une DSR plus longue augmentait de manière significative les taux de troponine, indiquant une LMPO, qui était associée à un pronostic à court terme moins favorable en termes d'événements cardiovasculaires. Cependant, d'autres facteurs, tels que la dysfonction rénale, plutôt qu'uniquement une DSR plus longue, sont également associés à une augmentation du taux de troponine.

Transcatheter aortic valve implantation (TAVI) is a well-established, effective, and safe treatment for severe aortic valve stenosis (AS).^1^^,^^2^ However, periprocedural myocardial injury (PMI), an acute complication of TAVI, remains a matter of concern. Elevated troponin level, indicative of myocardial injury, is a core diagnostic criterion for PMI according to the **V**alve **A**cademic **R**esearch **C**onsortium-**3** (VARC-3) criteria.^3^ VARC-3 defines PMI as postprocedural troponin level > 70-fold the upper limit of normal (ULN) or > 35-fold the ULN in the presence of new Q waves, left bundle branch block, flow-limiting angiographic complications, or myocardial loss on imaging.^3^ Although PMI is associated with worse long-term outcomes,^4^ its influence on short-term outcomes and its interaction with baseline patient conditions and procedural methods remain unclear.

Rapid pacing (RP), an essential component of TAVI, has been identified as the predominant factor that contributes to PMI diagnosis.^5^ RP is required for procedures such as percutaneous balloon aortic valvuloplasty (BAV) and precise deployment of balloon-expandable valves.^6^ During RP, the ventricular rate is intentionally increased to approximately 180 bpm, leading to systemic hemodynamic compromise, reduced microvascular tissue perfusion, and transient impairment of left ventricular systolic and diastolic functions, all of which may contribute to the development of PMI.^7^, ^8^, ^9^, ^10^

However, conflicting results have been reported regarding the relationship between RP and elevated troponin, a key biomarker of PMI.^6^^,^^11^^,^^12^ This discrepancy may be attributed to confounding factors such as renal dysfunction, advanced age, male sex, coronary artery disease (CAD), and reduced left ventricular ejection function (LVEF), all of which influence troponin levels.^13^, ^14^, ^15^ Notably, renal function plays a significant role in patients with an estimated glomerular filtration rate (eGFR) < 30 mL/min per 1.73 m^2^ (end-stage renal disease [ESRD]), who often exhibit approximately 3-fold higher cardiac troponin T (cTnT) levels than those with an eGFR > 60 mL/min per 1.73 m^2^.^13^^,^^14^ Therefore, accounting for or eliminating the effects of renal dysfunction is essential for accurately detecting clinically significant complications related to PMI.

Given these considerations, we aimed to evaluate the effect of RP on PMI, with a particular focus on PMI modification by renal dysfunction. In addition, we explored the impact of baseline patient conditions and types of implanted valves. We hypothesized that severe renal dysfunction significantly affects troponin levels after TAVI. Specifically, after excluding patients with ESRD, we compared the effect of rapid pacing time (RPT) on cTnT level elevation and PMI after TAVI. Furthermore, we examined the relationship between the duration of RPT and an accurately assessed increase in cTnT levels (ie, PMI), as well as short-term clinical outcomes.

## Methods

### Participant eligibility

**Figure 1:**
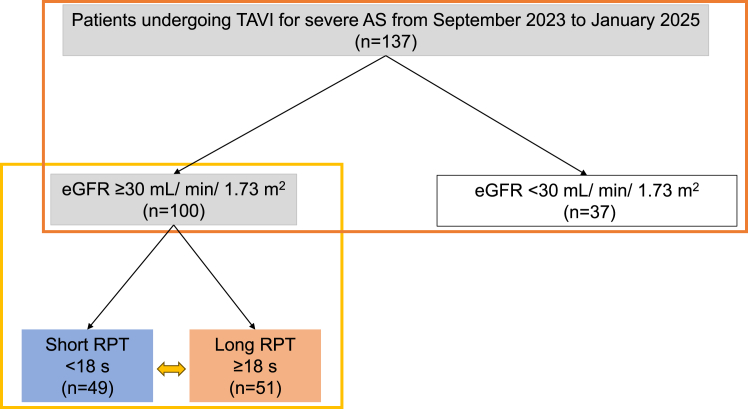
Flowchart of eligibility and study protocol. We investigated the association between baseline renal function and cTnT levels in 137 consecutive patients who underwent TAVI for AS at our hospital between September 2023 and January 2025 (**orange frame**). A total of 100 patients were included in the analysis, after excluding 37 patients with ESRD, defined as an eGFR < 30 mL/min per 1.73 m^2^ (**yellow frame**). Based on a mean RPT of 18 seconds, the patients were divided into 2 groups: short (n = 49) and long (n = 51) RPT groups. This division allowed us to investigate the associations of RPT with elevated cTnT levels, PMI, and short-term outcomes. AS, aortic stenosis; cTnT, cardiac troponin T; eGFR, estimated glomerular filtration rate; ESRD, end-stage renal disease; RPT, rapid pacing time; TAVI, transcatheter aortic valve implantation.

A total of 137 patients who underwent TAVI for severe AS at our hospital between September 2023 and January 2025 were included in this study (Fig. 1). Of these patients, 100 were enrolled in the study after excluding those with an eGFR < 30 mL/min per 1.73 m^2^ (n = 37). The patients were divided into short RPT (< 18 seconds) and long RPT (≥ 18 seconds) groups (n = 49 and 51, respectively), using the median RPT value of 18 seconds. No established threshold for determining long RPT exists in the context of TAVI, based on previous evidence or biological/physiological logic; hence, we used the data-driven median value as the threshold.

Our study was approved by the Clinical Research Appropriate Promotion Center at the Saitama Medical University International Medical Center (No. 2024-144).

### Data collection

Data on baseline characteristics, including age, sex, body surface area, and comorbidities (hypertension, dyslipidemia, diabetes mellitus, smoking habit, peripheral artery disease, stroke, active endocarditis, porcelain aorta, carotid artery stenosis, chest abnormalities, chronic obstructive pulmonary disease, malignancy, and immunodeficiency), were collected (Table 1). Additional clinical data collected included cardiovascular information (eg, previous heart surgery), electrocardiographic findings (rhythm, atrioventricular conduction, and intraventricular conduction), transthoracic echocardiographic findings (including LVEF and aortic valve status), and blood test results (including blood count and eGFR). Details of the TAVI procedure, including the approach and valve type, were documented (Table 2). Based on the VARC-3 criteria, cTnT levels were measured within 24 hours after TAVI.

**Table 1:** Baseline characteristics

	eGFR ≥ 30 mL/min per 1.73 m^2^ (n = 100)	Short RPT (n = 49)	Long RPT (n=51)	Short vs long RPT (*P* value)
Demographics				
Age, years	84.0 [81.0-87.0]	85.0 [81.5-88.0]	84.0 [79.0-87.0]	0.102
Men, n (%)	48 (48.0%)	26 (53.0%)	22 (43.1%)	0.605
BSA [mm^2^]	17.3 [14.4-20.47]	18.0 [14.7-20.8]	17.0 [14.4-19.9]	0.359
Risk factors				
Hypertension	86 (86.0%)	42 (85.7%)	44 (89.7%)	> 0.999
Dyslipidemia	41 (41.0%)	19 (38.7%)	22 (43.1%)	0.688
Diabetes mellitus	19 (19.0%)	12 (24.4%)	7 (13.7%)	0.109
Smoking	2 (2.0%)	0	2 (3.9%)	0.495
Peripheral artery disease	6 (6.0%)	3 (6.1%)	3 (5.8%)	0.715
Stroke	14 (14.0%)	8 (16.3%)	6 (11.7%)	0.573
Active endocarditis	1 (1.0%)	1 (2.0%)	0	0.490
Porcelain aorta	0	0	0	> 0.999
Carotid artery stenosis	2 (2.0%)	2 (1.5%)	0	0.237
Chest abnormalities	0	0	0	> 0.999
Chronic obstructive pulmonary disease	4 (4.0%)	1 (2.0%)	3 (5.8%)	0.617
Malignancy	18 (18.0%)	10 (20.4%)	8 (15.6%)	0.608
Immunodeficiency	6 (6.0%)	1 (2.0%)	2 (3.9%)	> 0.999
Cardiovascular information				
Previous heart surgery	8 (8%)	5 (10.2%)	5 (9.8%)	> 0.999
Previous PCI	8 (8.0%)	3 (6.1%)	5 (9.8%)	0.715
Previous PMI	3 (3.0%)	1 (2.0%)	2 (3.9%)	> 0.999
Coronary artery disease before TAVI	28 (28.0%)	16 (32.6%)	12 (23.5%)	0.596
Angina pectoris	6 (6.0%)	0	6 (11.7%)	0.026
Chronic myocardial infarction	11 (11.0%)	5 (10.2%)	6 (11.7%)	> 0.999
Arrhythmia	23 (23.0%)	11 (22.4%)	12 (23.5%)	> 0.999
Electrocardiogram findings				
Rhythm				
Sinus	85 (85.0%)	43 (87.7%)	42 (82.3%)	0.578
Atrial fibrillation	12 (12.0%)	5 (10.2%)	7 (13.7%)	0.760
Pacemaker	3 (3.0%)	1 (2.0%)	2 (3.9%)	> 0.999
Atrioventricular conduction				
First-degree atrioventricular block	5 (5.0%)	3 (6.1%)	2 (3.9%)	0.674
Intraventricular conduction				
CRBBB	10 (10.0%)	4 (8.1%)	6 (11.7%)	0.741
CLBBB	8 (8.0%)	6 (12.2%)	2 (3.9%)	0.155
Transthoracic echocardiography findings				
Left ventricular ejection fraction [%]	62.5 [47.0-73.0]	58.0 [44.0-72.0]	64.0 [52.0-75.0]	0.107
Aortic valve information				
Bicuspid	3 (3.0%)	2 (1.5%)	1 (1.6%)	0.613
Peak velocity [m/s]	4.1 [3.7-4.6]	4.1 [3.6-4.4]	4.1 [3.7-4.7]	0.534
Peak PG [mmHg]	67.2 [54.8-84.9]	67.2 [53.1-78.6]	67.2 [54.8-88.4]	0.537
Mean PG [mmHg]	39.9 [32.3-51.5]	39.9 [31.8-50.0]	40.0 [33.0-56.0]	0.463
AVA [cm^2^]	0.74 [0.61-0.90]	0.77 [0.61-0.91]	0.70 [0.61-0.90]	0.322
Aortic regurgitation (moderate or greater)	14 (14.0%)	6 (12.2%)	8 (15.6)	0.774
Mitral regurgitation (moderate or greater)	16 (16.0%)	6 (12.2%)	11 (21.5%)	0.288
Tricuspid regurgitation (moderate or greater)	11 (11.0%)	4 (8.1%)	7 (13.7%)	0.525
Left atrial diameter [mm]	43.0 [38.0-48.0]	42.0 [37.0-45.5]	44.0 [39.0-50.0]	0.070
IVST [mm]	12.0 [10.0-14.0]	12.0 [10.0-13.5]	12.9 [10.8-14.7]	0.086
PWT [mm]	12.0 [10.0-13.0]	11.0 [10.0-12.0]	12.0 [10.2-13.0]	0.157
LVDd [mm]	46.0 [41.0-48.7]	47.0 [43.4-49.8]	43.0 [40.0-48.0]	0.041
LVDs [mm]	28.0 [24.0-33.0]	29.5 [26.2-33.7]	27.0 [22.9-33.0]	0.042
Blood test findings				
White blood cells [× 1000/mm^3^]	5.8 [4.6-6.7]	5.94 [4.61-6.71]	5.62 [4.78-6.96]	0.636
Hemoglobin [g/dL]	11.8 [10.7-13.3]	12.0 [10.7-13.3]	11.8 [10.6-13.5]	0.919
Hematocrit [%]	36.1 [32.9-40.2]	36.0 [32.6-40.5]	36.3 [32.9-39.7]	0.873
Platelet [× 1000/mm^3^]	189.0 [159.3-220.5]	193.0 [164.0-221.5]	184.0 [144.0-221.0]	0.389
Albumin [mg/dL]	3.8 [3.4-4.0]	3.7 [3.3-4.1]	3.8 [3.4-4.0]	0.561
Creatinine [mg/dL]	0.87 [0.69-1.12]	0.91 [0.69-1.14]	0.80 [0.67-1.07]	0.434
PT-INR	1.00 [0.96-1.04]	0.99 [0.94-1.05]	1.00 [0.97-1.03]	0.282
eGFR [mL/min per 1.73 m^2^]	54.2 [43.6-67.7]	52.8 [43.8-69.9]	54.4 [43.0-67.1]	0.913

AVA, aortic valve area; BSA, body surface area; CLBBB, complete left bundle branch block; CRBBB, complete right bundle branch block; eGFR, estimated glomerular filtration rate; IVST, interventricular septum thickness; LVDd, left ventricular internal dimension in diastole; LVDs, left ventricular internal dimension in systole; PCI, percutaneous coronary intervention; PG, pressure gradient; PMI, pacemaker implantation; PT-INR, prothrombin time-international normalized ratio; PWT, posterior wall thickness; TAVI, transcatheter aortic valve implantation.

**Table 2:** Transcatheter aortic valve implantation procedure details

TAVI procedure	eGFR ≥ 30, mL/min per 1.73 m^2^ (n = 100)	Short RPT (n = 49)	Long RPT (n = 51)	Short vs long RPT *P* value
Approach				
Transfemoral	85 (85.0%)	43 (87.7%)	42 (82.3%)	0.926
Trans-subclavian	12 (12.0%)	6 (12.2%)	6 (11.7%)	0.946
Transapical	3 (3.0%)	0 (0%)	3 (5.8%)	0.348
Valve				
Self-expandable	55 (55.0%)	44 (80.0%)	11 (20.0%)	---
Predilation	48 (48.0%)	37 (77.0%)	11 (23.0%)	---
Inoue balloon	9 (9.0%)	9 (100.0%)	0	---
Balloon-expandable	45 (45.0%)	5 (11.1%)	40 (88.9%)	---
Predilation	38 (38.0%)	4 (10.5%)	34 (89.5%)	---
Inoue balloon	4 (4.0%)	3 (75.0%)	1 (25.0%)	---
RP				
Underwent RP	83 (83.0%)	32 (38.5%)	51 (61.4%)	---
Underwent predilation	86 (86.0%)	41 (47.7%)	45 (52.3%)	---
Total RPT during the entire procedure (s)	18.0 [9.0-28.7]	9.0 [0-12.0]	28.0 [24.0-35.0]	---

Data expressed as number (%) or as median [first to third quartile].

BAV, balloon aortic valvuloplasty; eGFR, estimated glomerular filtration rate; RP, rapid pacing; RPT, rapid pacing time; TAVI, transcatheter aortic valve implantation.

### Bioprosthetic valve selection and approach site

All patients who underwent TAVI had severe AS, and the optimal valve was selected based on the valve area, circumference, perivalvular structures, and vascular access, as assessed by preoperative electrocardiogram (ECG)-gated computed tomography (CT) scans and transthoracic echocardiography. The following bioprosthetic valves were used for TAVI: the balloon-expandable SAPIEN 3 Ultra Resilia (Edwards Lifesciences, Irvine, CA), the self-expanding CoreValve Evolut FX (Medtronic, Minneapolis, MN), and the self-expanding Navitor (Abbott Structural Heart, St. Paul, MN). Approach site selection was based on the preoperative ECG-gated CT scan and vascular ultrasonographic data (transfemoral, trans-subclavian, or transapical). RP was performed through right ventricular pacing in all cases. All balloon-expanding valves (BEVs) were deployed under rapid pacing at a rate of 180 bpm. All self-expanding valves (SEVs) were deployed without rapid pacing, using control pacing at 130 bpm. Even when predilation was performed, rapid pacing was not used if an Inoue balloon catheter (Toray, Tokyo, Japan) was used (Supplemental Fig. S1). Postdilation after TAVI valve deployment was not performed in any patient. RPT was defined as the total duration of RP during both predilation and valve deployment.

### Outcome measures

The primary endpoint was PMI/troponin levels, whereas the secondary endpoints were 30-day all-cause death and major adverse cardiovascular events (MACE), defined as a composite of cardiovascular death, stroke, new or worsening heart failure, and new pacemaker implantation. The incidence of each endpoint within 30 days of TAVI was evaluated. Uni- and multivariate Cox proportional hazards model were used for each outcome. The following multivariate analysis models were used to analyze the risk factors for all-cause mortality (Table 3):

• Model 1: Included common risk factors for all-cause death, including age and male sex.
• Model 2: Included risk factors related to cardiac death, including cTnT and CAD.
• Model 3: Included risk factors influencing cardiac death, including LVEF and eGFR.

**Table 3:** Uni- and multivariate Cox proportional regression analyses for predictive value for 30-day all-cause death and MACE

	Univariate analysis		Multivariate analysis	
	HR [95% CI]	*P* value	HR [95% CI]	*P* value
All-cause death model 1				
Age	0.021 [−0.146-0.185]	0.809	0.016 [−0.146-0.177]	0.847
Sex	−0.292 [−1.280-0.524]	0.488	−0.286 [−1.276-0.533]	0.498
All-cause death model 2				
ΔcTnT (× 1000)	1.669 [−0.303-3.341]	0.082	1.669 [−0.328-3.369]	0.085
CAD	−0.112 [−0.929-0.875]	0.792	0.001 [−0.001-0.003]	0.648
All-cause death model 3				
LVEF	−0.005 [−0.052-0.045]	0.808	0.997 [0.974-1.000]	0.158
eGFR	0.021 [−0.024-0.064]	0.340	0.021 [−0.024-0.064]	0.342
MACE model 1				
ΔcTnT (× 1000)	0.895 [0.363-14.62]	0.043	0.922 [0.414-1.563]	0.042
Male sex	0.270 [−0.744-0.339]	0.482	0.278 [0.680-0.437]	0.683
Age	0.056 [−0.137-0.080]	0.579	0.056 [−0.154-0.067]	0.424
MACE model 2				
ΔcTnT (× 1000)	0.895 [0.363-14.62]	0.043	0.907 [0.115-1.181]	0.043
LVEF	0.011 [−0.019-0.046]	0.490	−0.001 [−0.034-0.035]	0.924
CAD	0.278 [−0.695-0.426]	0.563	−0.021 [−0.615-0.666]	0.946
MACE model 3				
Hemoglobin	−0.127 [−0.466-0.199]	0.447	−0.202 [−0.546-0.145]	0.253
Approach site (transapical)	7.278 [−1.109-11.110]	0.099	7.282 [−1.121-11.228]	0.129
AVA	0.8306 [0.334-3.912]	0.828	1.905 [−0.518-4.153]	0.119

AVA, aortic valve area; CAD, coronary artery disease; CI, confidence interval; cTnT, cardiac troponin T; HR, hazard ratio; LVEF, left ventricular ejection fraction; MACE, major adverse cardiovascular events.

The following multivariate analysis models were used to analyze the risk factors for MACE:

• Model 1: Included risk factors for cardiovascular disease, including ΔcTnT, male sex, and age.
• Model 2: Included risk factors for cardiovascular disease, including ΔcTnT, LVEF, and CAD.
• Model 3: Included risk factors for cardiovascular disease, including hemoglobin, approach site (transapical), and aortic valve area.

### Statistical methods

Categorical variables are presented as number (percent) and continuous variables are presented as mean ± standard deviation for normally distributed data or as median [first to third quartile] for skewed data. The Mann-Whitney *U* test and the χ^2^ test were used to compare baseline characteristics between the short and long RPT groups. The relationship between baseline eGFR and cTnT levels was analyzed using simple regression. Kaplan-Meier analysis with the log-rank test was performed to compare 30-day all-cause mortality and MACE. Uni- and multivariate analyses were performed using Cox proportional hazards models to evaluate the associations between baseline variables, all-cause mortality, and MACE. The Wilcoxon matched-pairs signed-rank test was used to compare ΔcTnT between the short and long RPT groups and between the SEV and BEV. Similarly, the Wilcoxon matched-pairs signed-rank test was used to compare ΔcTnT between the non-RP and RP groups. The χ^2^ test was used to compare the proportions of PMI between the groups.

Statistical analyses were performed using JMP software (SAS Institute, Cary, NC) and GraphPad Prism software (GraphPad, San Diego, CA). Statistical significance was set at *P* < 0.05.

## Results

### Baseline characteristics and TAVI procedure details

Among the 100 patients included in the study, after excluding those with ESRD, the median age was approximately 84 years, with a nearly equal distribution of sexes (Table 1). Approximately 85% of the patients had a history of hypertension, and nearly 20% had a history of malignancy. CAD was identified in 28% of the patients prior to TAVI, and the median LVEF was preserved at approximately 62.5%. No significant differences were observed in most of the patient characteristics between the short and long RPT groups. However, significant intergroup differences were noted for history of angina pectoris (*P* = 0.026), left ventricular diameter in diastole (*P* = 0.041), and left ventricular diameter in systole (*P* = 0.042).

Regarding the details of the TAVI procedure, the access site for TAVI was trans-subclavian in 12% of patients, transapical in 1%, and transfemoral in the remaining patients (Table 2). The bioprosthetic valves used were almost evenly divided into SEVs and BEVs, with only 3 patients receiving the Navitor valve. Further, the proportion of self-expanding bioprosthetic valves was significantly higher in the short RPT group (*P* < 0.001) than in the long RPT group. No significant difference was observed for access site; however, the short RPT group exhibited a significantly higher proportion of SEVs (*P* < 0.001) and a significantly lower frequency of RP (*P* < 0.001) compared with the long RPT group. However, no significant intergroup differences were found for performance of BAV.

### Influence of renal function on baseline cTnT

**Figure 2:**
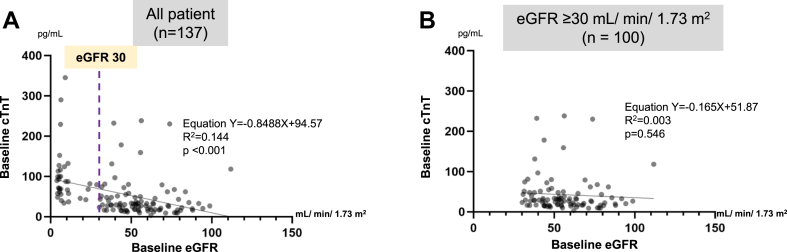
Influence of renal function on baseline cTnT. (**A**) A significant negative correlation was observed between baseline eGFR and cTnT in all 137 patients (*P* < 0.001). (**B**) Among the 100 patients, after excluding those with ESRD (eGFR ≥ 30 mL/min per 1.73 m^2^), no correlation was observed between baseline eGFR and cTnT. cTnT, cardiac troponin T; eGFR, estimated glomerular filtration rate; ESRD, end-stage renal disease.

A significant negative correlation was observed between baseline eGFR and cTnT levels in all 137 patients (*P* < 0.001) (Fig. 2A), but not in the subgroup of 100 patients after excluding those with ESRD (Fig. 2B).

### Impact of RPT on troponin elevation and PMI after TAVI

**Figure 3:**
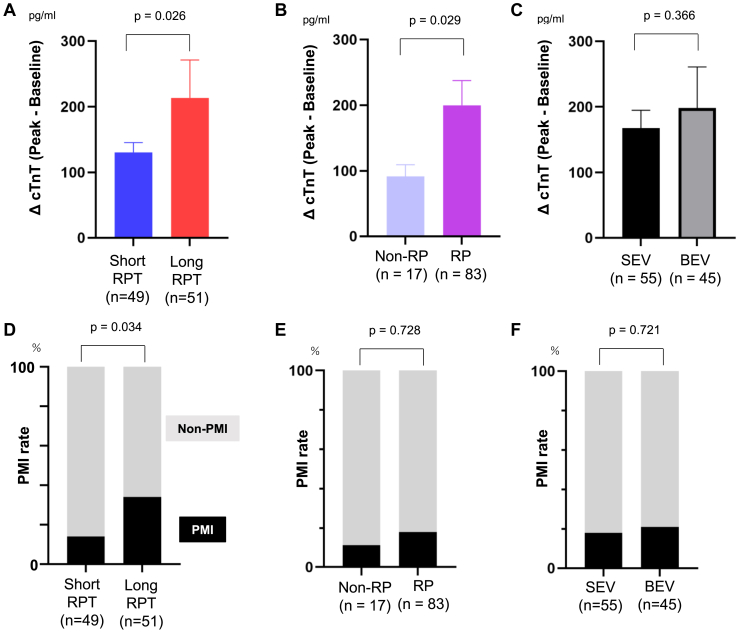
Impact of RPT on troponin elevation and PMI after TAVI. (**A**) Among the 100 patients, after excluding those with ESRD, the ΔcTnT (peak cTnT − baseline cTnT) after TAVI was significantly higher in the long RPT group than in the short RPT group (*P* = 0.026). (**B**) The RP group showed a significantly greater increase in ΔcTnT compared with the non-RP group (*P* = 0.029). (**C**) There was no significant difference in the increase of ΔcTnT between BEVs and SEVs. (**D**) The PMI rate was significantly higher in the long RPT group than in the short RPT group (14.2 vs 33.3, *P* = 0.034). (**E**) No significant difference was observed in PMI incidence between the non-RP and RP groups (11.7 vs 18.5, *P* = 0.728). (**F**) There was no significant difference in the rate of PMI between BEVs and SEVs (18.1 vs 17.7, *P* = 0.721). BEV, balloon-expanding valve; cTnT, cardiac troponin T; ESRD, end-stage renal disease; PMI, periprocedural myocardial injury; RP, rapid pacing; RPT, rapid pacing time; SEV, self-expanding valve; TAVI, transcatheter aortic valve implantation.

The long RPT group exhibited significantly higher ΔcTnT (peak baseline) levels after TAVI than the short RPT group (*P* = 0.026) (Fig. 3A). The RP group showed a significantly greater increase in ΔcTnT compared with the non-RP group (*P* = 0.029) (Fig. 3B). There was no significant difference in the increase of ΔcTnT between BEVs and SEVs (Fig. 3C). The PMI rate was significantly higher in the long RPT group vs the short RPT group (14.2 vs 33.3, *P* = 0.034) (Fig. 3D). The incidence of PMI did not differ significantly between the non-RP and RP groups (11.7 vs 18.5, *P* = 0.728) (Fig. 3E). There was no significant difference in the rate of PMI between BEVs and SEVs (18.1 vs 17.7, *P* = 0.721) (Fig. 3F).

### Kaplan-Meier analysis for all-cause death and MACE over 30 days

**Figure 4:**
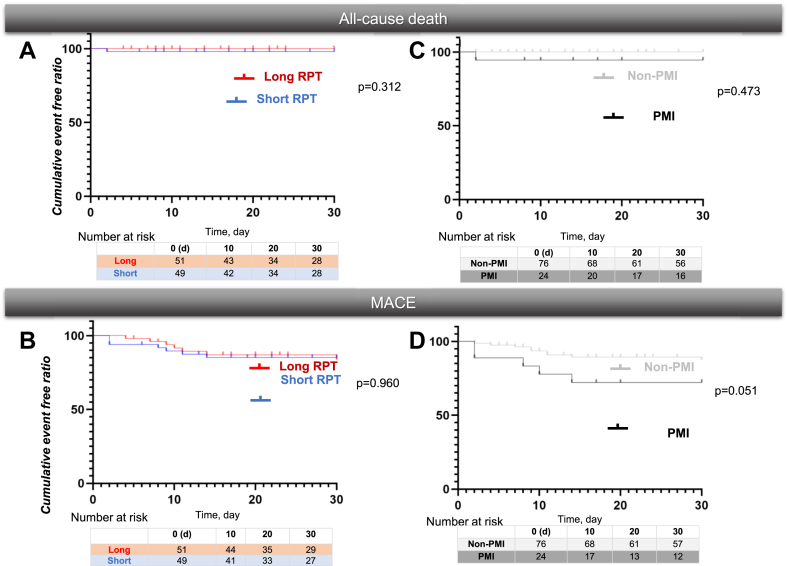
Kaplan-Meier analysis for all-cause death and MACE over 30 days. (**A, B**) No significant differences were observed in 30-day all-cause death and MACE between the short and long RPT groups. (**C**) There was no significant difference in overall 30-day mortality between groups with and without PMI. (**D**) The PMI group tended to have a higher likelihood of experiencing a 30-day MACE (*P* = 0.051). MACE, major adverse cardiac or cerebrovascular event; PMI, periprocedural myocardial injury; RPT, rapid pacing time.

No significant variation in 30-day all-cause mortality or MACE was found between the short and long RPT groups (Fig. 4, A and B). No significant difference was observed in 30-day all-cause mortality between the groups with and without PMI (hereafter referred to as PMI and non-PMI groups, respectively); however, the PMI group tended to have a higher likelihood of 30-day MACE compared with the non-PMI group (*P* = 0.051) (Fig. 4, C and D). Regarding short-term outcomes, the PMI group had 1 all-cause death (cardiac death) and 5 cases of MACE (1 cardiac death and 2 cases each of heart failure and pacemaker placement). In contrast, the non-PMI group had no cases of all-cause death and 2 cases of MACE (1 case each of heart failure and new pacemaker placement). ΔcTnT was identified as an independent prognostic factor for a 30-day MACE (*P* = 0.043) (Table 3).

## Discussion

### Main findings

In this study we evaluated 3 key aspects pertaining to patients undergoing TAVI for severe AS from the perspective of RPT: 1) renal function, 2) increased troponin levels and PMI development, and 3) short-term outcomes of PMI over 30 days (Central Illustration):

1. A significant and negative correlation was found between baseline eGFR and cTnT levels in the entire patient group (*P* < 0.0001) (Fig. 2). However, this association disappeared when patients with ESRD were excluded.
2. Among patients without ESRD, the long RPT group had a significantly higher ΔcTnT (*P* = 0.026) and PMI rate (14.2 vs 33.3, *P* = 0.034), compared with the short RPT group (Fig. 3).
3. The PMI group showed a trend toward more frequent 30-day MACE (*P* = 0.051) (Fig. 4D). In addition, ΔcTnT was identified as an independent prognostic factor for 30-day MACE (*P* = 0.043) (Table 3).

**Central Illustration:**
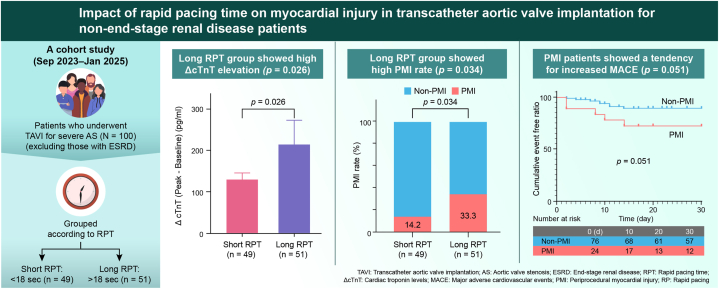
Impact of rapid pacing time on myocardial injury in transcatheter aortic valve implantation for non–end-stage renal disease patients. We analyzed data from 137 patients who underwent transcatheter aortic valve implantation (TAVI) between September 2023 and January 2025. The relationship between renal function and cardiac troponin T (cTnT) levels was assessed. To examine the impact of rapid pacing time (RPT) on cTnT elevation, periprocedural myocardial injury (PMI), and short-term outcomes, patients were grouped based on RPT duration. Among the 100 patients with an estimated glomerular filtration rate (eGFR) of ≥ 30 mL/min per 1.73 m^2^, 2 subgroups were defined: short RPT (< 18 seconds, n = 49) and long RPT (≥ 18 seconds, n = 51). The primary endpoint was PMI or cTnT levels, whereas secondary endpoints included 30-day all-cause mortality and major adverse cardiovascular events (MACE). A significant inverse correlation was found between eGFR and cTnT levels (*P* < 0.001). Patients in the long RPT group showed notably higher cTnT levels (*P* = 0.026) and a greater incidence of PMI (33.3% vs 14.2%, *P* = 0.034) compared with the short RPT group. No significant difference in 30-day outcomes was observed between the 2 RPT groups. However, patients with PMI tended to have a higher rate of MACE (*P* = 0.051). In addition, the change in cTnT (ΔcTnT) was an independent predictor of 30-day MACE (*P* = 0.043).

In a renal function–adjusted cohort after excluding patients with ESRD, excessive RPT was associated with significantly increased troponin levels and PMI incidence after TAVI, potentially increasing the risk of short-term cardiovascular events.

### Prolonged RP increases myocardial injury after TAVI

Troponin levels often increase after TAVI.^16^, ^17^, ^18^, ^19^ However, the mechanism underlying myocardial injury remains unclear.^5^ Potential causes include PMI due to hypotension during RP, mechanical trauma to the basal interventricular septum from the implanted bioprosthetic valve, microvascular embolization of plaque and valve debris, and paravalvular leakage.^17^^,^^19^, ^20^, ^21^, ^22^ The relationship between RP and post-TAVI troponin elevation has been inconsistently reported across studies,^12^^,^^23^^,^^24^ likely owing to confounding factors such as renal function.

In our cohort, after excluding those with ESRD, the long RPT group (defined by a median duration of 18 seconds) exhibited a significantly higher increase in cTnT levels after TAVI than the short RPT group (Fig. 4A). In addition, the expression of PMI after TAVI was significantly increased in the long RPT group (Fig. 4B). On the other hand, we observed a discrepancy wherein prolonged RPT was associated with elevated troponin levels but not with clinical outcomes. According to the VARC-3 definition of PMI, in addition to troponin elevation, clinical criteria---such as the presence of new Q waves, left bundle branch block, flow-limiting angiographic complications, or myocardial loss on imaging---are required. We believe this requirement may explain the observed gap in our results.

### Renal function and troponin elevation: implications for PMI assessment

Elevated troponin level after TAVI is indicative of PMI, and it has been associated with increased mortality and clinical events 1 year after the procedure.^25^, ^26^, ^27^ Therefore, precise evaluation of troponin, a key biomarker of PMI, is crucial. However, accurate assessment of troponin levels can be challenging because of the influence of various modifying factors.^13^, ^14^, ^15^ Renal function is considered to have a significant effect on troponin levels.^13^^,^^14^

We found a negative correlation between the baseline eGFR and cTnT levels (Fig. 2A). However, when patients with ESRD were excluded, no correlation was observed between the baseline eGFR and cTnT levels (Fig. 2B), suggesting that assessing cTnT levels in patients while excluding those with ESRD allows for a more accurate evaluation of PMI.

### Evolution of VARC criteria and impact of PMI on clinical outcomes

Since the establishment of the VARC criteria for defining PMI after TAVI in 2011, the current criteria have undergone revisions, evolving from VARC-2 to VARC-3 by 2021. The troponin elevation threshold, a key biomarker of PMI, was set at 15-fold the upper reference limit (URL) in VARC-2.^28^ However, in VARC-3, this threshold increased to 70-fold of the URL or 35-fold of the URL when combined with the clinical and ECG findings.^29^ This higher threshold enhances the precision of the PMI diagnosis and is expected to influence the impact of PMI on clinical outcomes. Under the previous VARC-2 criteria, studies have reported a correlation between PMI after TAVI and 1-year mortality.^25^^,^^30^ To date, only 1 study has addressed PMI after TAVI using the current VARC-3 criteria and identified an association between PMI and 1-year mortality.^4^

Patients with PMI showed a tendency toward an increased risk of 30-day MACE (Fig. 3D). Under VARC-2, only 1 study linked PMI to 30-day clinical outcomes.^30^ Future studies are expected to demonstrate the association between PMI and short-term clinical outcomes under the VARC-3 criteria. This shift is largely attributed to the higher troponin thresholds introduced in VARC-3, allowing for a more selective identification of PMI cases. In previous studies, concerns were raised that lower troponin thresholds in VARC-2 may have led to an overestimation of PMI.^31^ Our study has demonstrated a tendency toward an association between PMI and 30-day short-term clinical outcomes under the VARC-3 criteria, reinforcing the prognostic value of elevated troponin levels. These findings highlight the growing importance of PMI and its underlying cause, RP, in future management of TAVI.

### Limitations

Our study has some limitations. First, the sample size was relatively small. Larger multicenter studies are needed to further explore the relationship between RP and elevated troponin levels. However, our study is significant, because, to our knowledge, it is the first to demonstrate a correlation between RP and troponin elevation while excluding the influence of renal function.

Second, the observation period was short, and the long-term prognosis was not assessed. Therefore, further follow-up studies are warranted. Nevertheless, our ability to demonstrate the association between PMI and short-term prognosis using the revised VARC-3 criteria has important implications for the future perioperative management of TAVI.

## Conclusions

Using the revised VARC-3 criteria, prolonged RPT was strongly linked to higher troponin levels, reflecting increased PMI. Nonetheless, elevated troponin levels were also influenced by factors beyond RPT, including renal dysfunction.
