# Supplementary material for: Impact of Rapid Pacing Time on Myocardial Injury in Transcatheter Aortic Valve Implantation for Non–End-stage Renal Disease Patients
Source: CJC Open. 2025 Aug 28;7(12):1592–601. doi: 10.1016/j.cjco.2025.08.011 (PMC12800844; doi:10.1016/j.cjco.2025.08.011)
Supplement: Supplementary Figure 1 [file mmc1.pptx]

## Slide 1
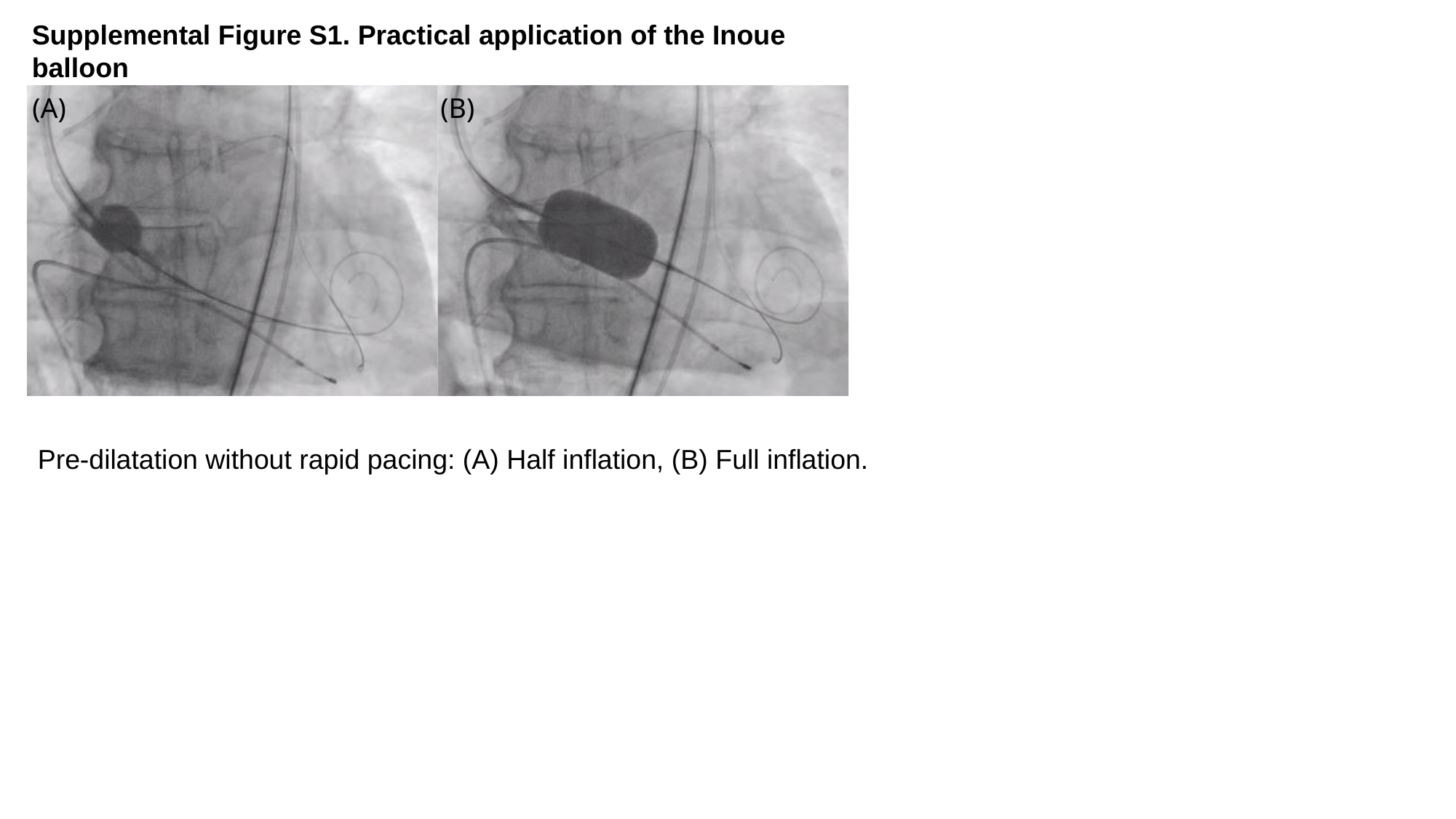

Supplemental Figure S1. Practical application of the Inoue balloon
(A)
(B)
Pre-dilatation without rapid pacing: (A) Half inflation, (B) Full inflation.
